# A qualitative study of the coverage of influenza vaccination on Dutch news sites and social media websites

**DOI:** 10.1186/1471-2458-13-547

**Published:** 2013-06-05

**Authors:** Birthe A Lehmann, Robert AC Ruiter, Gerjo Kok

**Affiliations:** 1 Department of Work & Social Psychology, Faculty of Psychology and Neuroscience, Maastricht University, P.O. box 616, 6200, MD Maastricht, The Netherlands

**Keywords:** E-health, Social media, Influenza, Influenza vaccination

## Abstract

**Background:**

Information about influenza and the effectiveness of vaccination against influenza is largely available on the Internet, and may influence individual decision making about participation in future influenza vaccination rounds. E-health information has often been found to be inaccurate, or even to contradict Health Authority recommendations, especially when it concerns controversial topics.

**Methods:**

By means of an online media monitoring programme, Dutch news sites and social media websites were scanned for the Dutch counterparts of the terms *influenza, vaccination, vaccine* and *epidemic* during February, March and April 2012. Data were processed with QSR NVivo 8.0 and analysed using a general inductive approach.

**Results:**

Three overarching themes were found in both media sources: (1) the (upcoming) influenza epidemic, (2) general information regarding the virus, its prevention and treatment, and (3) uncertainty and mistrust regarding influenza vaccination. Social media tended to report earlier on developments such as the occurrence of an influenza epidemic. The greatest difference was that in social media, influenza was not considered to be a serious disease, and more opposition to the flu shot was expressed in social media, as compared to news media.

**Conclusions:**

News media and social media discussed the same topics regarding influenza, but differed in message tone. Whereas news media reports tended to be more objective and non-judgmental, social media more critically evaluated the harmfulness of influenza and the necessity of the flu shot. Media may influence decision making and behaviours of Internet users and may thereby influence the success of vaccination campaigns and recommendations made by health authorities. Social media may be more of a problem in this sense, since it is neither controlled nor censored. Future research should investigate the actual impact of Internet media on the influenza decision making process of its users.

## Background

The Internet is an important source for accessing health information. In fact, 55% of Internet users search for health information online [1-3]. The Internet is the largest and most easily accessible library in the world and enables users to find information in a time-saving way. By the year 2000, more than 70 000 e-health websites existed [4]. E-health websites are websites that provide health information for educational purposes, self-care, and for the simplification of health-communication [5]. The Internet is the second most trusted source of health information, following the personal advice of one’s own general practitioner [6,7]. However, concerns have been raised by medical professionals and Internet users about the quality and comprehensibility of health information, especially when it is directed at the broader public [8,9]. That is, the Internet is an unregulated resource that not only enables anyone to access information, but also makes it possible for anyone to publish information [6,10]. The information overload is enormous [9], which makes it increasingly difficult to ensure the credibility of health information sources. Users mostly use search engines like Google and Yahoo to access health websites. However, a study by Berland and colleagues shows that searches with a search engine require high reading abilities and often do not lead to websites with relevant content [8]. Moreover, a number of different studies found website content did not adhere with official recommendations for prevention and treatment [9]. For example, in a study about the reliability of websites that informed parents about home management of an ill child, only 4 out of 41 websites displayed information in accordance with official recommendations [11].

After consulting health information on the Internet, people feel reassured twice as much as they feel anxious [12]. This could mean that people select sources on the basis of what they already believe to be true. This also means that people who already distrust public health recommendations may be biased to read information that is given by providers holding the same attitude. In social psychology, this tendency of people to favour information that confirms their belief has been termed the confirmation bias, a bias which can occur unintentionally and without awareness [13].

### Social media

Social media refers to Internet content that is continuously modified by all collaborating users of a publicly accessible website instead of by professionals [14]. It offers easy ways for people to share and read information on a large scale. In contrast to social media websites, news sites refer to websites that generate original news written exclusively by registered members (e.g., editorial staff).

Social networking has increased rapidly in recent years, making Twitter and Facebook the most popular platforms for sharing and communicating information [15]. More than half of all Internet users joined a social networking site in 2009 [16]. It has been found that people trust the opinions of their peers more than they trust the opinions of officials, which makes social networking sites increasingly powerful [15,17]. Individuals turn to each other for advice and trust that the experiences of their friends and acquaintances represent the truth [15]. It has been found that health information in online support groups and chat rooms is often inaccurate [9]. Social networking sites, such as Twitter and Facebook may show a similar insufficiency regarding health information. There has been, however, no study as of yet investigating the content of health information on social networking sites.

The purpose of this study is to explore the content of health information regarding influenza vaccination in the month before, during, and after an influenza epidemic occurred in the Netherlands. Cline and Haynes [9] have suggested that using the Internet for accessing health information is dangerous for already controversial topics. Influenza vaccination is a highly controversial issue in the Netherlands that is heavily discussed in the news media and also in the social media.

Objectivity is not always guaranteed and this might especially be true for social media platforms in which no censoring takes place. This could in turn negatively influence the decision making and behaviour of Internet users towards influenza vaccination and thereby may influence the success of Health Authority recommendations with regard to influenza vaccination. Therefore, the purpose of this study is to describe the news site and social media website content about influenza vaccination on the Internet, as well as the similarities and differences between these two types of media content.

## Methods

### Data collection

In this article, we describe the Dutch Internet content of news sites and social media websites that became accessible in the month before, during, and after the 2012 influenza epidemic occurred. We retrospectively selected the three months February (before), March (during) and April (after), based on official documentations of influenza activity in the Netherlands by the Netherlands Institute for Health Services Research (NIVEL).

Data were collected using *Clipit*[18], an online media monitoring programme that can be used to search the Internet for preselected terms. In the case of this study, the search terms *influenza*, *vaccination*, *vaccine* and *epidemic* were monitored daily in more than 10.000 Dutch Internet sources, including news sites and press reports, discussion forums, weblogs, newsletters, reviews, as well as social media websites. A search profile with the aforementioned terms was already activated in June 2011 by the National Institute for Public Health and the Environment (RIVM).

News websites, as defined by Clipit, range from print media that is additionally represented on the Internet to more unconventional websites for members of groups that share certain beliefs, such as the belief that vaccinations are harmful. Social media websites monitored by Clipit were Twitter, Facebook, Linkedin and Hyves. Hyves is the Dutch equivalent to Facebook. In the case of social networking sites, only reports that were made publicly accessible by their authors were detected by the monitoring programme. Clipit continuously provided links to websites in which one or more of the above mentioned search terms were used. These links were individually opened and the content of the appearing Internet site was then copied and transferred to Word documents. Only the first page that appeared when opening the link was taken into consideration and read in its entirety. No further links were opened, thus restricting the level of analysis to the primary source. Data collection and analysis was performed by the first author.

### Data analysis

The Internet content was processed with QSR NVivo 8.0 (Doncaster, Australia). The content analysis was based on a general inductive approach [19] and conducted by a single coder (BAL; see Author’s Contributions). Through detailed reading of the raw data, themes were identified and data were subsequently categorized under separate headings (called nodes in QSR NVivo 8.0). Afterwards, categories were linked to one another, which led to the identification of overarching themes relevant for the description of online content regarding influenza as a whole. This process was repeated separately for each month and for the two different kinds of online media sources (news sites and social media websites). Exclusion criteria were website content about influenza-related topics concerning countries other than the Netherlands (including the Dutch speaking part of Belgium) and website content about the bird flu. Additionally, themes on news sites and in social media posts that only occurred once, or showed no consistency with more often reported themes were excluded to reduce the overload of information. Following analysis, quotes were selected on the basis of their representativeness for the findings and were subsequently translated from Dutch into English.

## Results

In February, March and April 2012, three different overarching themes were consistently identified in the online content of news sites and social media websites: (1) the (upcoming) influenza epidemic, (2) general information regarding the virus, its prevention and treatment, and (3) the uncertainty and mistrust regarding influenza vaccination in the Netherlands. The information is summarized below according to these three themes and per month. In total, 3552 of the 4441 reports that were detected by Clipit were included in the results: 1305 reports (n = 204 on news websites; n = 1101 on social media websites) in February, 1527 reports (n = 276 on news websites; n = 1251 on social media websites) in March and 721 reports (n = 61 on news websites; n = 660 on social media websites) in April (see Table 1). See Table 2 for an overview of the information that is summarized below.

**Table 1:** Number of included and excluded influenza reports for each month and total

	**Total reports**	**News media**			**Social media**		
**Month**	**Total (%)**	**Total**	**Incl. (%)**	**Excl. (%)**	**Total**	**Incl. (%)**	**Excl. (%)**
Reports detected	4441	939	541 (58)	398 (42)	3502	3012 (86)	490 (14)
Febr	1574 (35)	299	204 (68)	95 (32)	1275	1101 (86)	174 (14)
March	1992 (45)	480	276 (58)	204 (42)	1512	1251 (83)	261 (17)
April	875 (20)	160	61 (38)	99 (62)	715	660 (92)	55 (8)

**Table 2:** Summary of website content by theme, month and source

**Month**	**Epidemic**	**Information, prevention & treatment**	**Uncertainty & Mistrust**
**News sites & press**			
**Febr 2012**	Influenza activity low; increase in other European countries; criteria for epidemic; identifying the type of influenza.	Difference between flu and common cold; symptoms of flu; possible complications of an infection; variants of virus; severity of influenza; health effects of vaccination; vaccinated women and higher birth weight; prevention and treatment.	Uncertainty about effectiveness of vaccination; difficulty of proving effectiveness; collaboration between science, politics and pharmaceutical industry; dangerous effects of vaccination; spreading illness; narcolepsy as effect of the pandemic (H1N1) vaccine in 2009.
**March 2012**	Epidemic in south of the Netherlands; presence of epidemic; recognition of the flu; effectiveness of flu shot; complications for risk groups; decreasing influenza activity.	Only 70% of employees stayed at home when sick, possibly because of the economic crisis; information about virus and vaccine; Google flu; prevention, treatment and vaccination as the only effective protection.	Narcolepsy; collaboration between science and the pharmaceutical industry.
**April 2012**	End of epidemic; criteria for epidemic identification.	One nursing home with 30% infections.	Narcolepsy; loss of vaccines as a result of inaccurate storage.
**Social media**			
**Febr 2012**	South of Netherlands closer to epidemic threshold (*links to news sites*); epidemic present (*user experiences/ perceptions*).	Difference between stomach flu and seasonal flu, viral and bacterial infection; Prevention (e.g. vitamin C&D); effectiveness flu shot (*user experience*).	Uncertainty about effectiveness of vaccination; ineffectiveness flu shot (*user experience + links to websites*); advice against vaccination.
**March 2012**	Late onset epidemic (*links to news sites*); presence of epidemic (*links to news sites + user experience*).	40% Dutch people have flu each year; less flu-related absenteeism because of economic crisis; work overload in hospitals during epidemic; vitamin D metabolism and flu; prevention; effectiveness flu shot (*own experience*).	Possibility of getting flu despite flu shot; economic background vaccination; ineffectiveness of flu shot; dangerousness of flu shot (e.g. Alzheimer, narcolepsy); collaboration between science and pharmaceutical industry.
**April 2012**	Epidemic present (*own experience*); end of epidemic (*links to news sites*).	Numbers of deaths epidemic 2012 (*link to website*); decreasing work absenteeism; effectiveness of flu shot (*own experience*).	Possibility of getting flu despite flu shot; ineffectiveness + dangerousness of flu shot; hygiene in workplace as means of prevention; discussion about loss of vaccines as a result of inaccurate storage.

### News websites

#### February 2012

##### Upcoming epidemic

A number of news sites reported about the fact that influenza activity was low in the Netherlands and that epidemics usually occur in December or January. Some articles discussed the possibility that 2012 might be a year without an influenza epidemic:

“It is possible that the flu won’t peak until the end of February. That is the same time that primary schools have vacation and many people will go on a winter holiday, which increases the chance that the flu may even pass us by this year.” (http://www.nursing.nl)

In the beginning of February 2012, reports showed that most European neighbors experienced similar levels of influenza activity. However, in the middle of February, news sites reported an increase in influenza activity in the south of Europe. This resulted in an increase of news reports about the threat of an upcoming epidemic in the Netherlands.

“In the past weeks, flu became more active in more and more countries around us; In South and South-East Europe, as well as in Norway. Meanwhile, the flu reached Belgium, but did not yet reach Germany, Britain and Denmark.” (http://www.gezondheidskrant.nl)

Next to information about the development of influenza activity, news sites also informed readers about the process by which influenza epidemics are identified:

“NIVEL (Netherlands Institute for Health Services Research) speaks about an epidemic if within two successive weeks more than 51 per 100.000 people with flu-like conditions are reported and virological research reveals the virus in nose- and throat samples.” (gezondheid.blog.nl)

Other reports discussed the type of virus that was expected in 2012:

“[…] it is mainly influenza B and influenza AH3N2. Both flu-variants are covered by the vaccine that was offered for high risk groups in the winter season. The AH1N1-virus that caused the pandemic in 2009 is rarely seen.” (medischcontact.artsennet.nl)

Several times it was indicated in news site reports that there was no apparent reason for a late influenza epidemic.

##### Information about the virus, prevention and treatment

Next to information about the expected upcoming influenza epidemic, readers were informed about the differences between the flu and a common cold, symptoms of the flu, possible complications, especially for high risk groups, and the effect of influenza vaccination. The mutations that occur in the influenza virus were outlined in several articles as a means to explain why annual vaccination is required:

“No single influenza virus is the same. Every year it is a different virus. You can get the flu every year. Therefore, the vaccine has a different composition every time. The composition is matched to the viruses that are expected to be present the following winter. Therefore, it is necessary to get vaccinated every year.” (mens-en-gezondheid.infonu.nl)

A number of reports emphasized the severity and consequences of influenza:

“The flu is the most underestimated illness that exists.” (http://www.healthylives.nl)

“Every year approximately 820.000 Dutch people get the flu. During an average influenza epidemic in the winter, 250 to 2.000 people in the Netherlands die because of the flu or complications of the flu. Victims mainly belong to the high risk groups.” (http://www.nivel.nl)

Recent research was also discussed on news sites. For example, a study was reported describing the finding that mothers who got vaccinated against influenza while they were pregnant delivered babies with a higher birth weight than did mothers who did not get vaccinated against influenza [20].

Next to general information, means of prevention and treatment of an influenza infection were described. Some articles dealt with homeopathic means to prevent and treat influenza but also indicated that this is an additional tool next to vaccination. For example:

“People that got vaccinated in autumn are protected, but what can people do that did not get vaccinated to protect themselves? […] For people that want to protect themselves but dread getting a flu shot, there is Polyinfluenzium.” (http://www.nieuwslog.nl)

Other reports discussed ways of keeping or acquiring a good physical resistance, such as drinking a lot of water, wearing warm clothes, eating healthily, ensuring good hand hygiene, and performing outdoor physical activities.

##### Uncertainty & mistrust

News sites also dealt with uncertainty and mistrust regarding science. In particular, the uncertainty surrounding whether influenza vaccination is effective or possibly even dangerous was discussed. One report about the ‘Evidence Beast’ summarized the debate that was then described in the content of a number of news sites that were published online in February. It deals with the difficulty in proving that the morbidity and mortality rate would be much higher if influenza vaccination did not exist or had not been effective.

“We have to prove that by doing something good, something bad will not happen, which would have happened if we had not done anything. Sounds complicated and that is what it is. […] We have learned this from the flu vaccination incident.” (http://www.skipr.nl)

Some news reports discussed the developments in the Netherlands with regard to the views that the general population hold about science. One issue is that a proportion of the general public does not see science as an independent and reliable source anymore, when it might have direct consequences for their own lives. People are said to fear that science is biased by politics, as well as by the pharmaceutical industry.

“Citizens don’t just accept the ‘expert stories’, even more so, when they have direct consequences for their lives. When scientists and politics get too close, problems can arise […].” (http://www.volkskrant.nl)

There were several articles reporting about the dangerous effects that influenza vaccination might have. These reports provide assumed evidence for the fatal effects of vaccination. Individual ingredients and their effect on one another are described. Supposed “experts” are cited and speak about secret plans of the government to deliberately infect people with a virus through the vaccine in order to create an elite or to control the growth of the world’s population.

“Recently, it (flu vaccination) is used more and more to spread illness. That is the reason why most people who get the flu in winter are the same people who get the flu shot regularly.” (http://www.argusoog.org)

Other articles strengthened their point of view by referring to the increased incidences of narcolepsy in children and its link to the pandemic (H1N1) vaccination in 2009, which was first suggested by a Finnish study [21]. Readers are asked to be more critical about vaccinations and to propagate their opinion to other people in order to prevent fatal consequences.

“We hope that you, the reader of this article, will do your best to NOT keep quiet. That during gatherings, such as parties and dinners you will NOT avoid this topic, but instead try to convince people that vaccines are NOT safe. It is a FACT!! There are enough scientists who strongly doubt the necessity of vaccines.” (http://www.wanttoknow.nl)

#### March 2012

##### (Upcoming) epidemic

In the beginning of March, several news sites reported that there was an on-going epidemic in the south of the Netherlands:

“The Netherlands did manage to avoid the flu for a long period this winter. In the south of the Netherlands, however, there has been an epidemic since March 1^st^.” (http://www.dichtbij.nl, http://www.omroepzeeland.nl, http://www.drimble.nl)

It was also indicated that the chance for the north of the Netherlands to experience an epidemic had increased. More people were identified with the influenza virus and schools were about to open again after the vacation. However, in the north of the Netherlands, activity was still below the epidemic threshold.

“In the week of February 27^th^ until March 4^th^, on average 50 per 100.000 Dutch people reported having flu-like complaints to their general practitioner. […] Last week it was 47. We are actually very close to the epidemic threshold.” (gezondheid.blog.nl)

After the first week of March, news sites mostly reported that the epidemic was now in fact happening in the Netherlands.

“In the beginning of March 2012, the Netherlands did experience an influenza epidemic after all. More than 78 per 100.000 inhabitants went to the general practitioner with flu-like complaints. For a start it is a mild epidemic. Most people get better after 3–5 days – says NIVEL, the Netherlands Institute for Health Services Research.” (http://www.amstelveenweb.com)

Several articles additionally provided information about how to recognize whether someone was actually infected with the influenza virus. A few articles also referred to the wider impact of an influenza epidemic and the effectiveness of the flu shot.

“The flu is an illness that will go away by itself, so people don’t have to worry. […] The flu shot provides you with approximately 70 percent protection against the flu. People who get the flu shot and get ill anyway are still better protected against complications from the virus.” (Donker, NIVEL, http://www.nrc.nl, http://www.AD.nl, http://www.volkskrant.nl, http://www.gezondheidsnet.nl)

It was also mentioned that young children, aged 0 – 4 years of age, as well as the elderly, aged 65 years of age and older, are at highest risk to suffer from complications of an influenza infection. In the fourth week of March, news sites started to report about the end of the epidemic as influenza activity decreased again.

“The influenza epidemic – as far as you can call it such – seems to already be on its way out.” (gezondheid.blog.nl)

Influenza activity in the Netherlands was said to be comparable to the activity in Europe with regard to the time of occurrence, as well as the type of virus that was detected.

##### Information about the virus, prevention and treatment

The most often reported news regarding general information about influenza in March was the discovery that in the past winter only 70% of employees decided to stay at home when ill with flu instead of going to work. Readers were advised to stay at home.

“Real flu is not to be taken too lightly. Sometimes complications can emerge, such as a sore throat, pneumonia, meningitis, inflammation of the myocardial muscle, as well as additional bacterial infections. […] Therefore, it is important to recover completely.” (http://www.fmm.nl)

The economic crisis was seen as a possible reason for this phenomenon. Next to these news items, there were also some articles with general information about the virus and the process by which the vaccine works. Other articles dealt with the idea of using Google searches in order to predict epidemics, a project called Google Flu.

Discussions regarding means of prevention and treatment of influenza were similar to those reported in February. Readers were informed how to improve their health status with rest and enough sleep, a lot of water, healthy food and outdoor physical activity. As a means of prevention, a number of articles stated that the flu shot was the only effective protection. This was also the conclusion of an article that examined the effectiveness of other flu medication.

“According to pharmacist X, those medications (to treat flu complaints) do not help to prevent someone from getting the flu. […] The only proved, effective method of prevention is vaccination.” (http://www.rtl.nl)

##### Uncertainty and mistrust

As in February, in March there were a number of articles concerned with the finding that narcolepsy was found more often in children after the pandemic (H1N1) vaccination in 2009. Furthermore, debates about the trust in science and the collaboration between scientists and the pharmaceutical industry were again discussed in online news. Consequences of disabling the interaction between science and the industry were discussed.

“Well yes, maybe the paid experts are too positive about medication, but you could also say that medical practitioners who do not collaborate with the industry do not do so because they are too negative about medication. […] If we have the illusion that medicine gets better if we stop the interactions, […] then there will simply not be any new medication.” (http://www.artsennet.nl)

#### April 2012

##### End of epidemic

In April 2012, several news sites reported that the epidemic of March had already ended.

“The influenza epidemic was short and not even strong. In the beginning of this year, the flu stayed for some weeks beneath the threshold of an epidemic. Not until the beginning of March did the flu start to spread. However, the peak only lasted one week.” (http://www.nerderland-davos.nl)

Readers were again informed about the criteria that are used to identify an epidemic.

##### Information about the virus, prevention and treatment

Some news sites reported about a nursing home in which 45 of the 160 residents had had a serious influenza infection, 12 of whom had to be hospitalized. However, the situation was resolved after a few days and further transmission was stopped.

“The flu breakout put a lot of pressure on the care in the (nursing home). Director X: ‘Suddenly, a quarter of the residents got ill. The organization was in chaos for a short while, because a lot of extra care had to be given. […]’.” (http://www.hoogeveen.nu)

##### Uncertainty and mistrust

Most articles dealing with uncertainty that were published in April discussed the finding that there was now more evidence that narcolepsy is caused by Pandemrix, the vaccine used for the pandemic (H1N1) in 2009, as well as a news report about an employee of the National Vaccine Institute of the Netherlands that had left those vaccines out of the refrigerator, at great financial cost.

“THE HAGUE – Pandemic (H1N1) 2009 vaccines that could have been sold abroad, were made useless by a blunder of an employee of the National Vaccine Institute (NVI) in Bilthoven.” (http://www.rtl.nl)

### Social media websites

#### February 2012

##### (Upcoming) epidemic

In the beginning of February 2012, posts on social networking sites mostly reported that influenza activity was still low and that there was still no epidemic present in the Netherlands. However, it was reported that the south of the country was getting closer to epidemic levels. Several posts on Twitter provided a link to corresponding news site reports.

“The flu epidemic is not yet present in the Netherlands, but it seems to be coming. (link to news site)“ (Twitter)

In February, there were also already posts that an epidemic had arrived in the Netherlands. People who put the posts on Twitter referred to their own experience and news they had read, without providing the corresponding article.

“The flu epidemic is a fact: Half of my floor is ill including myself #stomach flu” (Twitter)

“What is happening to me? My throat aches. I read that there is a flu epidemic in the Netherlands.” (Twitter)

##### Information about the virus, prevention and treatment

On some social networking sites, information about the virus was concerned with the difference between stomach flu and the seasonal flu, as well as the fact that flu is caused by a virus rather than a bacterial infection.

“Stomach flu, right? A flu shot won’t help with that because it's not really the flu.” (Twitter)

Regarding prevention of the flu, there were several posts containing advice like going outside enough, getting enough rest, eating fruit and taking vitamins. Some Tweets suggested that vitamin D and C are effective in preventing the flu.

“Additional vitamin C! Vitamin C is a real virus killer. It has to be a high dose, a minimum of 3000 mg per day. Can be more during flu.” (Twitter)

There were several posts with links to news sites with prevention tips and information about the flu. Many posts on social networking sites were about the flu shot, of which several expressed belief in the effectiveness of the flu shot in preventing influenza.

“Luckily I got vaccinated and the variant is included (in the flu shot). Luckily, I won’t get the flu!” (Twitter)

People either informed other people about their own positive experience with the flu shot, or reported that the flu shot had at least resulted in weaker flu symptoms.

“No, it’s fine. I think X had a small bout of flu, but thanks to the flu shot it was very weak.” (Twitter)

Several people stated that they were planning to get the flu shot next time in order to prevent illness, which they were currently experiencing.

“I will see whether I can get the flu shot next year!! Again affected by one or another virus.” (Twitter)

##### Uncertainty and mistrust

There were also several posts expressing uncertainty regarding the flu shot. People who did not take the flu shot themselves wondered whether they should have taken it.

“The doctor also says that I should get the flu shot, but is it true? I strongly doubt it.” (Twitter)

The majority of posts about the flu shot expressed doubt over its effectiveness in preventing the flu, and thought that it might even cause flu.

“Since I had the flu shot in November, I’ve had a double pneumonia and a strong cold. Went through the pain of the flu shot for nothing.” (Twitter)

Others copied a link to a study that concluded that there is no evidence for the effectiveness of influenza vaccination.

“Summarized: 5700 studies about influenza vaccination, 31 were found to be done well and no clear evidence that influenza vaccination makes sense.” (Twitter)

Several people stated that they did not believe in the effectiveness of the flu shot and advised others to not get vaccinated.

“Getting injected with diseases artificially, never start with that. Such a shot makes you ill in order to build up antibodies, but if you have the flu rarely you shouldn’t get such a shot.” (Facebook)

“Flu vaccination? Don’t do it!!!” (Twitter)

Advice to not get vaccinated also seemed to influence uncertainty regarding influenza:

“Get the flu shot this year after everything I’ve heard from others about their experiences?” (Twitter)

#### March 2012

##### (Upcoming) epidemic

In March 2012, there were some posts about the late flu epidemic on Twitter and Facebook. Most posts included links to public health websites or news sites. Some posts were written by representatives of the National Institute of Public Health and the Environment (RIVM).

“X@RIVM: two things stand out about the influenza epidemic: it is mainly influenza A H3N2 and it has not been this late in 25 years.” (Twitter)

Several posts, including news site links, indicated that the epidemic was expected to start soon in the Netherlands.

“There is a good chance that a national flu epidemic will strike this week. The north of the Netherlands was almost flu-free the past weeks, however with the end of the Easter vacation in this area, chances of infection will now increase.” (Facebook)

In the beginning of March, there were several posts about the presence of an influenza epidemic, mostly including links to news sites. Furthermore, some people who posted about feeling ill received reactions that there was an epidemic in the Netherlands.

X: “I am feeling ill. Lying in my bed since 9 o’clock, then cold, then again really hot…and I’m complaining. :-p #stomach ache #headache #sigh” – Y: “Influenza virus is going around. Get well soon!” (Twitter)

##### Information about influenza, prevention and treatment

In March, several posts on Twitter referred to a website with statistics saying that approximately 40% of all Dutch people have the flu each year.

“Flu epidemic close: annually, approximately four out of ten Dutch people get the flu (link to website with statistics).” (Twitter)

Some social media posts informed the reader about the finding that there was less flu-related absenteeism from work, possibly because of the economic crisis.

“Less flu-related absenteeism because of the crisis (link to news site).” (Twitter)

At the same time, a number of Twitter users stated that news reports about the flu epidemic would give employees a reason for absenteeism.

“Employers watch out, the newspapers think it is necessary to give everyone a reason to stay at home. There seems to be a flu epidemic.” (Twitter)

Readers were also presented with links to reports about hospitals, which were said to have had a lot of extra work during the epidemic.

Several posts indicated a possible connection between metabolism of vitamin D and the flu.

X: “There is no causal evidence for influenza virus - > flu. Vitamin D metabolism is […] a far more logical explanation.” – Y: “Vitamin D and influenza – Wikipedia (link) summary: influenza virus is bullshit, flu is a seasonal illness caused by a lack of Vitamin D.” (Twitter)

In addition, people wondered why others worry about the on-going epidemic.

X: “Why the fuck should you be worried about the flu epidemic? It is the FLU! Get over yourself!” – Y: “Sure, in the past 100 years only more than 20 million people died because of it. No big deal.” – X: “That’s what I mean. The flu you are suffering from during an epidemic is the same flu you would get without an epidemic.” (Twitter)

Regarding flu prevention, a number of posts offered links to websites and summarized corresponding tips on how to stay healthy, including eating healthily, exposure to the sun, and wearing functional sportswear. Furthermore, vitamin D as a means of preventing flu infection was discussed again.

“The flu can be shortened or prevented with a high dose of vitamin D, but this is not possible with the flu shot.” (Twitter)

Other posts expressing uncertainty were concerned with the question of whether “a mild epidemic” means that the epidemic is mild or that the flu is mild.

“You are wondering ‘there is a mild flu epidemic in the Netherlands’… is it the flu that is mild or is it the epidemic?!” (Twitter)

Twitter users also asked whether the symptoms they were experiencing were normal for flu.

“I’m worried: does the recent flu have side-effects such as dizziness and prickling in hands and feet? I’ve had it for a week already…” (Twitter)

Some people stated that they were avoiding the flu shot because of its possible long term consequences.

As in February 2012, several posts on Twitter expressed belief in the effectiveness of the flu shot in preventing influenza. Several people stated that they had had good experiences with the flu shot and advised others to get the flu shot as well.

“What about the flu shot? Did you think about that yet? It works really well for me. Since taking the shot I’ve had no cold and wasn’t ill anymore. #tip” (Twitter)

Others stated that they were planning to get the flu shot next time.

“I think I will get a flu shot next year. (Ill again)” (Twitter)

People belonging to the risk groups also posted about their annual flu shot.

##### Uncertainty & mistrust

In March, uncertainty that was expressed on social media websites dealt with several different topics regarding influenza vaccination. Again, several people expressed uncertainty about the possibility of getting the flu in spite of being vaccinated.

“Oh yes, does a flu shot mean that you indeed can’t get the flu anymore? That is what I’m wondering about.” (Twitter)

Additionally, it was questioned whether it is a good idea to get vaccinated against influenza and if there are economic reasons for being invited to get the flu shot.

“Already flu complaints since 3 weeks; does the flu shot help or is it just an auxiliary income for general practitioners?” (Twitter)

Again, the majority of posts about the flu shot dealt with the ineffectiveness of it in terms of preventing the flu. People posted about their own or about others’ experiences in this regard.

“I only know people who got really ill because of or despite of the flu shot.” (Twitter)

Several posts discussed the harmful effects of the flu shot, including links about the flu shot causing Alzheimers and narcolepsy. Narcolepsy was thought to be caused by the pandemic (H1N1) flu shot from 2009.

“Mysterious sleep disease affects 50.000 Germans! Link to flu shot suggested in Finland. (link to website).” (Twitter)

Another related link was introduced with “All the reasons why you should not get the flu shot this year” on Twitter. Some people expressed that it was more or less a matter of luck whether one is protected by the flu shot or not.

X: “Why do I always get the flu that isn’t included in the flu shot????” – Y: “Because the flu shot only protects you against half of the flu variants. ” (Twitter)

Some posts again indicated that there are economic reasons involved in the vaccination recommendations, which made them question the necessity of vaccination.

“It is getting more obvious that politics regarding vaccination are influenced by the principle ‘the one who pays, decides.’ (link) #flu shot” (Twitter)

Some posts discussed the collaboration between science and the pharmaceutical industry and questioned the reasons for this collaboration.

“The flu is a threat to our health, therefore, every year the flu shot has to be taken. Result: extra millions for the pharmaceutical industry and the state’s finances! Explain to me why we Dutch people have to save millions?????????????????” (Facebook)

#### April 2012

##### (End of) epidemic

In April 2012, several Twitter users reported either that they themselves felt ill or that a lot of people around them were ill, and wondered whether a flu epidemic was occurring.

“I have the flu…I guess I’m in good company #epidemic? #at home” (Twitter)

Several posts informed readers that the flu epidemic of 2012 was over again and that it was comparably short.

“Flu News: Flu epidemic finished earlier than in other years: ‘The flu epidemic had a rather short duration.’ (Link to news site)” (Twitter)

##### Information about the virus, prevention and treatment

Several times, links to websites providing statistics with regard to the flu were posted with information about the number of deaths.

“Two thousand additional deaths during the cold wave in February and the flu in March. (link to website with statistics)” (Twitter)

Furthermore, it was suggested that absenteeism should decrease again as a result of the end of the epidemic. In a number of Twitter-conversations, people advised each other to take time to get better after a flu infection.

X: “Working through it is unreasonable, get well first.” – Y: “You are right X; the flu will always disturb it; better to get additional rest and then hopefully get back into shape afterwards.” (Twitter)

In April, several Twitter posts again expressed the belief that the flu shot is effective in preventing an infection or that it will at least weaken the symptoms of the flu. Furthermore, people informed others via Twitter that they were planning to get the flu shot next time.

“Maybe I have to get the flu shot every year.” (Twitter)

##### Uncertainty & mistrust

In April 2012, there were again also posts that expressed uncertainty about the possibility of getting the flu despite having had the flu shot.

“Really, are that many people ill?? I actually can’t be ill because of the flu shot, but I am ill anyway, weird.” (Twitter)

Most people that posted something about the flu shot reported being ill because of or despite of the flu shot.

“For the first time in my life I got a flu shot! For the first time in years I have the FLU!!!” (Twitter)

In addition, several posts speculated as to whether the flu shot is effective, or could actually be harmful to one’s health.

“In this article, AGAIN the proof of the fact that vaccines don’t work and are not well studied before they are given to thousands of people.” (Facebook)

Furthermore, one link was posted a number of times that cites a Danish scientist, concluding that employers waste money with paying for the flu shot for their employees.

“Employers could better spend their money on improving hygiene in the workplace instead of on flu shots for their employees. (link to website)” (Twitter)

Moreover, a discussion on social media sites was visible about a news report that an employee of the National vaccine institute in 2009 had forgotten to put pandemic (H1N1) vaccines back in the refrigerator. As a result, 1,2 million vaccines were rendered useless.

“We never should have bought them in the first place. Fear. ‘Millions of flu vaccines made useless through blunder’ (link to news site).” (Twitter)

Views were expressed that a ban on any pay rise for two years was not an appropriate punishment for the mistake made by the employee concerned. Some posts provided links to news sites reporting that the RIVM and the minister of health of the Netherlands did not agree with the information that was given in the original press report. It was said to be exaggerated.

“RIVM: False article about destruction of flu vaccines in “national newspaper” (link to website)” (Twitter)

## Discussion

The present study aimed to describe the online content of news sites and social media websites across a period of three months in which an influenza epidemic occurred in the Netherlands. By means of an online media monitoring programme, more than 10.000 Dutch websites, including social media were scanned for the search terms *influenza*, *vaccination*, *vaccine* and *epidemic* during February, March and April 2012. Three different overarching themes were consistently identified: (1) the influenza epidemic, (2) more general information about the virus, prevention and treatment, and (3) uncertainty and mistrust regarding influenza vaccination.

With regard to content, news sites mainly reported about the progress of the influenza epidemic, the criteria for detecting an epidemic, and the type of virus that was identified in 2012. Most of the described content was neutral and informative and matched across different news sites. In line with our findings, Wright and Hinson [16] found traditional news media to be more accurate, credible, ethical and truth-telling more often than social media. On social media websites, there were links to the same news sites, also reporting that a flu epidemic was approaching or already happening in the Netherlands. The difference was that on social media websites, the information that an epidemic was occurring was reported earlier than on news sites. This was mostly supported by user perceptions. At the same time, when news sites already reported the end of the epidemic, there were still posts on Twitter from users wondering whether there was a flu epidemic. This indicates that some people, instead of searching for news on the Internet, preferred to ask about this information on social media websites. This is in accordance with a notion outlined by Qualman, who suggested that Internet users often trust the opinion of their peers more than that of officials [15]. Furthermore, the earlier appearance of information about influenza activity on social media websites is in accordance with research stating that certain information is registered earlier via social media sites than by official registration attempts [22,23].

More general information about the influenza virus was also provided on news sites. This included explanations about differences between influenza and a common cold, symptoms of the flu, its seriousness, and possible complications, especially for young children and the elderly. Readers were further informed that the flu shot is the most effective means of prevention, but that there are also homeopathic remedies and that good physical health is important in preventing an infection. Moreover, readers could see that people are less likely to stay at home when they have been ill in the past years, possibly because of the economic crisis. Social media websites offered similar information about different kinds of infections. However, while these were stated as facts in news media, in social media this kind of information was subject to discussion. Additionally, advice for prevention of the flu was given, mostly supported by links to websites that offer advice. In contrast to what appeared on news sites, on social media sites vitamin D and C were often named as important for physical resistance to infections.

In the last category, uncertainty and mistrust, news sites and social media content both dealt with the debate about the collaboration between science, politics and the pharmaceutical industry. A topic that was discussed on more unconventional news sites was the possible dangerous consequences of flu vaccination, including conspiracy theories about controlling population growth and illnesses that are said to be caused by vaccines. On social media websites, flu was often said not to be a serious disease and there was a considerable amount of criticism regarding the flu shot. It was claimed that the flu is often taken as an excuse to not work. The belief that the national recommendations to get vaccinated were mostly driven by economic reasons was also expressed several times.

On social media websites, links to news media are posted and contents of those news sites are discussed. Additionally, as suggested by Asur and Huberman [23], it is noticeable that several news sites, as well as public health websites, use social media to promote and spread information. It can be seen that especially on Twitter, links to news sites are shared and then re-shared by other people. As a result, information spreads quickly on a “many-to-many global platform” that is also used as a tool by a number of news sites and public health websites [15].

One difference especially visible between news sites and social media websites was that the majority of news sites reported that influenza vaccination is the most effective or only effective means of preventing influenza infections. Whereas on social media websites, the majority of messages concerning the flu shot expressed views that the flu shot is not effective and may even be dangerous to one’s health. Furthermore, whereas messages on social media websites that report that the flu shot is effective are mostly reports of one’s own experience with the shot, anti-vaccination messages are often supported with links to supposedly scientifically proven articles from less objective websites. This is of concern, as it is conceivable that people who already distrust influenza vaccination will possibly feel their views to be confirmed by what they read and people who are unsure about the flu shot may be persuaded by what they are told is scientific evidence about vaccination. Moreover, across the three months that we monitored social networking sites, anti-vaccination messages were put online twice as much as pro-vaccination messages. Messages expressing uncertainty about influenza vaccination were present just as much as pro-vaccination messages. News media, on the other hand, presented information mainly in favour of influenza vaccination.

This study has been executed by means of a qualitative method. Therefore, the findings are descriptive in nature and do not enable us to make causal inferences about them, nor indicate relative importance of the different themes that emerged. Data collection and analysis was performed by only the first author. Not including a second coder could have biased the results and made it impossible to apply inter-rater reliability. It must be noted further that the distinction between the two Internet sources we described was based on the categorization that is made within the online media monitoring programme Clipit. As a result, news websites included a wide range of different websites that generate original reports. These include print media that is additionally represented online, public health websites, but also more unconventional websites for people holding specific opinions. We decided not to further distinguish between those sources, because we think it is reasonable to believe that e-health users are directed to news about influenza and influenza vaccination in this broad sense or decide to engage in these topics on the social networking site of their choice. In addition, we were not able to obtain information on the number of followers and the characteristics of the readership. This information could have been helpful in stratifying the findings further in terms of importance and target group relevance.

## Conclusions

News media and social media show some important similarities, as well as differences. The overarching themes identified on news sites and social media websites are roughly the same. However, particular topics seem to appear earlier on social networking sites, such as the occurrence of an influenza epidemic. Influenza is evaluated differently by the two media sources. In social media, influenza is often said not to be a serious disease. With regard to influenza vaccination, it is noticeable that there is considerably more criticism expressed on social media websites than on news sites. However, there are also a number of news sites that contradict Health Authority beliefs about the necessity of influenza vaccination. This might influence the success of vaccination campaigns and Health Authority vaccination recommendations. This study is a first step in identifying the importance of e-Health in the formation of an opinion with regard to influenza vaccination. Future research should explore the specific impact of online media on decision making and actual behaviour with regard to health information.

## Abbreviations

RIVM: National Institute for Public Health and the Environment; NIVEL: Netherlands Institute for Health Services Research.

## Competing interests

All authors declare that they have no competing interests.

## Authors’ contributions

BAL, RACR and GK contributed to the conception, design of the study, as well as the interpretation of the data. BAL acquired the data and undertook analysis. All authors contributed to drafting the paper and read and approved the final manuscript.

## Pre-publication history

The pre-publication history for this paper can be accessed here:

http://www.biomedcentral.com/1471-2458/13/547/prepub

## References

[B1] Eysenbach G Köhler C Health-related searches on the internet JAMA 2004 291 2946 10.1001/jama.291.24.2946 15213205

[B2] Hsu J Huang J Kinsman J Fireman B Miller R Selby J Ortiz E Use of e-health services between 1999 and 2002: a growing digital divide JAMIA 2004 12 164 171 15561786 10.1197/jamia.M1672 PMC551548

[B3] Brodie M Flournoy RE Altman DE Blendon RJ Benson JM Rosenbaum MD Health information, the internet, and the digital divide Health Aff 2000 19 255 265 10.1377/hlthaff.19.6.255 11192412

[B4] Grandinetti DA Doctors and the web: help your patients surf the net safely Med Econ 2000 77 28 34 10848409

[B5] Oh H Rizo C Enkin M Jadad A What is eHealth (3): A systematic review of published definitions J Med Internet Res 2005 7 e1 15829471 10.2196/jmir.7.1.e1 PMC1550636

[B6] Dolan G Iredale R Williams R Ameen J Consumer use of the internet for health information: A survey of primary care patients Int J Consum Stud 2004 28 147 153 10.1111/j.1470-6431.2003.00363.x

[B7] Dumitru RC Bürkle T Potapov S Lausen B Wiese B Prokosch H-U Use and perception of internet for health related purposes in Germany: results of a national survey Int J Public Health 2007 52 275 285 10.1007/s00038-007-6067-0 18030943

[B8] Berland GK Elliott MN Morales LS Algazy JI Kravitz RL Broder MS Kanouse DE Muñoz JA Puyol J-A Lara M Watkins KE Yang H McGlynn EA Health information on the internet, Accessibility, quality, and readability in English and Spanish JAMA 2001 285 2612 2621 10.1001/jama.285.20.2612 11368735 PMC4182102

[B9] Cline RJW Haynes KM Consumer health information seeking on the internet: the state of the art Health Educ Res 2001 16 671 692 10.1093/her/16.6.671 11780707

[B10] Hardey M ‘E- health’: the internet and the transformation of patients into consumers and producers of health knowledge Inf Commun Soc 2001 4 388 405

[B11] Impicciatore P Pandolfini C Casella N Bonati M Reliability of health information for the public on the World Wide Web: systematic survey of advice on managing fever in children at home Br Med J 1997 314 1875 1879 10.1136/bmj.314.7098.1875 9224132 PMC2126984

[B12] Andreassen HK Bujnowska-Fedak MM Chronaki CE Dumitru RC Pudule I Santana S Voss H Wynn R European citizens’ use of E-health services: a study of seven countries BMC Publ Health 2007 7 53 59 10.1186/1471-2458-7-53 PMC1855923 17425798

[B13] Nickerson RS Confirmation bias: a ubiquitous phenomenon in many guises Rev Gen Psychol 1998 2 175 220

[B14] Kaplan AM Haenlein M Users of the world, unite! The challenges and opportunities of social media Bus Horiz 2010 53 59 68 10.1016/j.bushor.2009.09.003

[B15] Qualman E Socialnomic. How social media transforms the way we live and do business 2011 Ipswich, MA: Business Book Summaries

[B16] Wright DK Hinson MD An updated look at the impact of social media on public relations practice Public Relat J 2009 3 1 27

[B17] Schmitt-Beck R Mass communication, personal communication and vote choice: the filter hypothesis of media influence in comparative perspective Brit J Polit Sci 2003 33 233 259

[B18] Clip it online media monitoring http://www.clipit.nl/

[B19] Thomas DR A general inductive approach for analyzing qualitative evaluation data Am J Eval 2006 27 237 246 10.1177/1098214005283748

[B20] Steinhoff MC Omer SB Roy E El-Arifeen S Raqib R Dodd C Breiman RF Zaman K Neonatal outcomes after influenza immunization during pregnancy: a randomized controlled trial Can Med Assoc J 2012 184 645 653 10.1503/cmaj.110754 22353593 PMC3314035

[B21] Partinen M Saarenpää-Heikkilä O Ilveskoski I Hiblin C Linna M Olsén P Nokelainen P Alen R Wallden T Espo M Rusanen H Olme J Sätilä H Arikka H Kaipainen P Julkunen I Kirjavainen T Increased incidence and clinical picture of childhood narcolepsy following the 2009 H1N1 pandemic vaccination campaign in Finland PLoS One 2012 7 e33723 10.1371/journal.pone.0033723 22470463 PMC3314680

[B22] Marquet RL Bartelds AI Van-Noort SP Koppeschaar CE Paget J Schellevis FG Van der-Zee J Internet-based monitoring of influenza-like illness (ILI) in the general population of the Netherlands during the 2003–2004 influenza seasons BMC Publ Health 2006 6 242 249 10.1186/1471-2458-6-242 PMC1609118 17018161

[B23] Asur S Huberman BA Predicting the future with social media 2010 Arxiv preprint arXiv:1003.5699

